# Improving Diagnostic Accuracy in Food Allergy

**DOI:** 10.1016/j.jaip.2020.09.037

**Published:** 2021-01

**Authors:** Ru-Xin Foong, Jennifer A. Dantzer, Robert A. Wood, Alexandra F. Santos

**Affiliations:** a Faculty of Life Sciences and Medicine, Department of Women and Children's Health (Paediatric Allergy), School of Life Course Sciences, King's College London, London, United Kingdom; b Children's Allergy Service, Evelina London Children's Hospital, Guy's and St. Thomas' NHS Foundation Trust, London, United Kingdom; c Division of Allergy, Immunology, and Rheumatology, Department of Pediatrics, Johns Hopkins University School of Medicine, Baltimore, Md; d Peter Gorer Department of Immunobiology, School of Immunology and Microbial Sciences, King's College London, London, United Kingdom; e Asthma UK Centre for Allergic Mechanisms of Asthma, London, United Kingdom

**Keywords:** Food allergy, Diagnosis, Skin prick test, Specific IgE, Basophil activation test, Mast cell activation test, BAT, Basophil activation test, CMA, Cow's milk allergy, CRD, Component-resolved diagnostics, EPIT, Epicutaneous immunotherapy, LBA, Luminex-based assay, LTP, Lipid-transfer protein, MAT, Mast cell activation test, NPV, Negative predictive value, OFC, Oral food challenge, OIT, Oral immunotherapy, PPV, Positive predictive value, ROC, Receiver operating characteristic, sIgE, Specific IgE, SPT, Skin prick test

## Abstract

The diagnosis of food allergy can have a major impact on the lives of patients and families, imposing dietary restrictions and limitations on social activities. On the other hand, misdiagnosis can place the patient at risk of a potentially severe allergic reaction. Therefore, an accurate diagnosis of food allergy is of utmost importance. The diagnosis of food allergy is often established by the combination of the clinical history and allergen-specific IgE; however, without a clear history of an allergic reaction, the interpretation of IgE sensitization tests can be difficult. There are also rare cases of clinical food allergy in the absence of IgE sensitization. For that reason, testing for suspected food allergy ideally requires access to oral food challenges (OFCs), which are currently the gold standard tests to diagnose food allergy. As OFCs are time consuming and involve the risk of acute allergic reactions of unpredictable severity, the question remains: how can we improve the accuracy of diagnosis before referring the patient for an OFC? Herein, we review the predictive value of different tests used to support the diagnosis of food allergy, discuss implications for therapy and prognosis, and propose a diagnostic approach to be applied in clinical practice.

**Information for Category 1 CME Credit**

Credit can now be obtained, free for a limited time, by reading the review articles in this issue. Please note the following instructions.

**Method of Physician Participation in Learning Process:** The core material for these activities can be read in this issue of the Journal or online at the *JACI: In Practice* Web site: www.jaci-inpractice.org/. The accompanying tests may only be submitted online at www.jaci-inpractice.org/. Fax or other copies will not be accepted.

**Date of Original Release:** January 1, 2021. Credit may be obtained for these courses until December 31, 2021.

**Copyright Statement:** Copyright © 2021-2023. All rights reserved.

**Overall Purpose/Goal:** To provide excellent reviews on key aspects of allergic disease to those who research, treat, or manage allergic disease.

**Target Audience:** Physicians and researchers within the field of allergic disease.

**Accreditation/Provider Statements and Credit Designation:** The American Academy of Allergy, Asthma & Immunology (AAAAI) is accredited by the Accreditation Council for Continuing Medical Education (ACCME) to provide continuing medical education for physicians. The AAAAI designates this journal-based CME activity for 1.00 *AMA PRA Category 1 Credit*™. Physicians should claim only the credit commensurate with the extent of their participation in the activity.

**List of Design Committee Members:** Ru-Xin Foong, MBBS, Jennifer Dantzer, MD, MHSc, Robert A. Wood, MD, and Alexandra F. Santos, MD, PhD (authors); Scott H. Sicherer, MD (editor)

**Learning objectives**:

1. To describe the diagnostic performance profile (ie, sensitivity and specificity) of skin prick test and specific IgE to allergen extracts.
2. To describe the information provided by individual allergen components and allergen epitopes of specific foods.
3. To explain the advantages of the basophil and mast cell activation tests compared with skin prick test and specific IgE when diagnosing food allergy.
4. To indicate the ideal sequence of allergy tests to optimize accuracy and reduce oral food challenges when assessing patients with suspected food allergy.

**Recognition of Commercial Support:** This CME has not received external commercial support.

**Disclosure of Relevant Financial Relationships with Commercial Interests:** R. Foong declares no relevant conflicts of interest. J. Dantzer receives research support from National Institute of Allergy and Infectious Diseases (NIAID). R. A. Wood receives research support from NIAID, Aimmune, Astellas, DBV, HAL-Allergy, Regeneron, and Sanofi; and royalties from UpToDate. A. F. Santos reports grants and personal fees from Medical Research Council (MR/M008517/1); grants from Asthma UK, the National Institute for Health Research through the Biomedical Research Centre (BRC) award to Guy's and St Thomas' NHS Foundation Trust, the Immune Tolerance Network/NIAID/National Institutes of Health; personal fees from Thermo Scientific, Nutricia, Infomed, Novartis, Allergy Therapeutics, and Buhlmann; and research support from Buhlmann and Thermo Scientific through a collaboration agreement with King's College London. S. H. Sicherer declares no relevant conflicts of interest.

The diagnosis of food allergy can have a major impact on the lives of patients and their families, imposing dietary restrictions and often limitations on social and family activities. On the other hand, misdiagnosis can place the patient at risk of a potentially severe allergic reaction. Therefore, an accurate diagnosis of food allergy is of utmost importance. The clinical history is key for an accurate diagnosis, and in cases with a clear history of typical IgE-mediated symptoms after the ingestion of a specific food, the diagnosis can be relatively straightforward. However, diagnosis is much more challenging in the absence of a clear history of an allergic reaction or of uneventful consumption of an age appropriate amount of the allergenic food.

The diagnosis of IgE-mediated food allergy requires evidence of IgE sensitization by skin prick test (SPT) or serum IgE. IgE sensitization is more common than clinical food allergy and there are also rare cases of clinical food allergy in the absence of IgE sensitization. The diagnosis is often established by the combination of the history and allergen-specific IgE; however, without a clear history of an allergic reaction, the interpretation of IgE sensitization tests can be difficult. For that reason, testing for suspected food allergy ideally requires access to an oral food challenge (OFC), which is currently the gold standard test to diagnose food allergy. However, as OFCs are time consuming and involve the risk of acute allergic reactions of unpredictable severity, the question remains: how can we improve the accuracy of diagnosis before referring the patient for an OFC?

## Skin Prick Test and IgE to Allergen Extracts

Skin prick testing and specific IgE (sIgE) levels are diagnostic modalities used to detect the presence of sIgE antibodies to the food source.^1^ Positive results have traditionally been considered an SPT wheal diameter of ≥3 mm greater than the negative control or an sIgE ≥0.35 kU/L. However, a positive result alone indicates sensitization and does not equate to food allergy.^2^ Using the 3 mm and 0.35 cutoffs, SPT and sIgE have high sensitivity and negative predictive value (NPV), but low specificity and positive predictive value (PPV), and thus may lead to overdiagnosis.^2^^,^^3^ Studies have demonstrated that a larger SPT wheal size and/or higher sIgE correlates with increased likelihood of clinical allergy.^1^^,^^2^^,^^4^ Hence, specificity can be increased by using available 95% PPVs (Table I), but then sensitivity is reduced.

**Table I:** Diagnostic cutoffs for specific IgE and skin prick testing with 95% positive predictive

Foods	Specific IgE^1^^,^^5^, ^6^, ^7^, ^8^		Skin prick test^1^^,^^6^^,^^9^
	95% PPV	50% NPV	95% PPV
Cow's milk∗	15 kU/L (32 also reported) Infants ≤2 y: 5 kU/L	2 kU/L	≥8 mm Infants ≤2 y: 6 mm
Egg∗	7 kU/L Infants ≤2 y: 2 kU/L	2 kU/L	≥7 mm Infants ≤2 y: 4-5 mm
Peanut	15-34 kU/L	2 kU/L if history of reaction 5 kU/L is no history of reaction	≥8 mm Infants ≤2 y: 4 mm
Fish	20 kU/L	–	
Tree nuts	20 kU/L		≥8 mm for walnut ≥12 mm for cashew
Sesame	50 kU/L (86% PPV)		≥8 mm

*NPV*, Negative predictive value; *PPV*, positive predictive value.

∗ These numbers were derived from uncooked milk and direct egg and do not apply to baked milk or baked egg.

Although these cutoffs can be very useful in diagnosing food allergy, they have many limitations. These tests alone are by no means definitive, and it is not uncommon for patients to tolerate a food even with markedly elevated SPT and sIgE results.^10^ It is also important to consider that diagnostic cutoff values have varied widely in different studies, presumably due to differences in the patient populations and disease prevalence.^3^ SPT and sIgE thresholds may be dependent on age, but there is limited information in children <2 years.^5^ It is also important to note that the level of positivity does not predict reaction threshold or severity.^11^ Finally, many individuals have intermediate range results that fall between the NPV and PPV value where a definitive diagnosis is difficult and an OFC is often needed.^12^

A recent study reviewed 1247 OFCs and calculated SPT and sIgE receiver operating characteristic (ROC) thresholds for almond (12 mm, 12.2 kU/L), cashew (4.5 mm, 1.2 kU/L), egg (13 mm, 9.6 kU/L), hazelnut (7 mm, 14.6 kU/L), milk (8 mm, 20.1 kU/L), peanut (9 mm, 10.7 kU/L), pecan (7 mm, 1.8 kU/L), sesame (11 mm, 7.5 kU/L), walnut (4 mm, 13.5 kU/L), and wheat (5.5 mm, 43.1 kU/L) based on subjects with a positive OFC to a maximum cumulative dose of 500 mg.^13^ At defined SPT values, the PPV was 100% for all tested foods except pecan, which was 95%. The sIgE thresholds had a PPV of 64% for sesame, 73% for hazelnut, and ≥95% for the other included allergens. In addition, Saf et al^14^ reviewed outcomes of 341 OFCs to sesame and diagnostic accuracy of SPT and sesame sIgE. The median cumulative eliciting dose to which patients reacted was 500 mg of sesame protein. An SPT wheal size ≥6 mm was the optimal cutoff in the ROC curve, with a specificity of 87% and a sensitivity of 54%. A 95% PPV was not found, but was estimated using modeling to be ≥14 mm. Twelve patients had a negative SPT and a positive OFC. Interestingly, sesame sIgE was higher in patients who passed a sesame OFC versus failed (2.15 vs 1.88 kU/L). On the contrary, Sokol et al^15^ found that sesame-allergic subjects had a higher sesame sIgE than those who were sesame tolerant (median 31.2 vs 2.2 kU/L) and concluded that sIgE was not predictive of the outcome of the sesame OFC. The different conclusions from these studies could be due to different OFC dosing and different populations, namely the fact that the study from Mount Sinai only included patients who had been referred for challenges and in the latter study only a minority of patients were challenged.

## Specific IgE to Allergen Components

Component-resolved diagnostics (CRD) measures IgE to specific proteins within a food.^16^ This testing aims to better characterize the sensitization profile to determine clinically significant sensitization compared with clinically irrelevant cross-reactivity. Storage seed proteins (such as Ara h 2) are far more likely to be associated with true clinical reactivity. Profilins, Bet v 1 homologs, and PR-10 are pollen-cross reactive components associated with pollen-food allergy syndrome. The lipid-transfer proteins (LTP) family and less so PR-10 can be associated with systemic reactions and pollen cross-reactivity.

IgE to Ara h 2 (storage protein) and to a much lesser extent Ara h 1, Ara h 3, Ara h 6, and Ara h 9 are the most important predictors of symptomatic peanut allergy.^17^^,^^18^ Ara h 8 is a Bet-v 1 homolog and can indicate pollen-food syndrome. Like for sIgE to total peanut extract, recommended Ara h 2 cutoffs vary widely by study (0.35-42.2 kU/L had 90%-95% PPV) (Table II).^10^^,^^18^^,^^23^ Using the sensitization profile can help characterize risk and who should undergo an OFC.

**Table II:** Proposed diagnostic cutoff levels and positive predictive value (PPV) for specific IgE to individual allergen components

IgE to individual allergen components	IgE (kU_A_/L)	PPV
Milk casein to diagnose baked milk allergy^19^	20.2	69%
Egg ovomucoid		
to diagnose baked egg allergy^20^	50	95%
to diagnose cooked egg allergy^21^	26.6	95%
to diagnose raw egg allergy^22^	5.21	95%
Peanut Ara h 2^10^^,^^18^^,^^23^	0.35-42.2	90%-95%
Hazelnut Cor a 9^23^, ^24^, ^25^	1-2	79%-100% specificity
Hazelnut Cor a 14^23^^,^^26^	0.72-47.8	87%-90% specificity
Cashew Ana o 3^27^^,^^28^	0.162	98% specificity95%
Soya Gly m 8 ^29^	13.55	89%74%
Wheat Tri a 19^30^^,^^31^	0.040.41	100%81%

Commercially available hazelnut component testing includes sIgE toward Cor a 1 (PR-10), Cor a 8 (LTP), and Cor a 9 and Cor a 14 (storage proteins). Cor a 9 and Cor a 14 are associated with systemic reactions. Various diagnostic cutoffs have been proposed.^3^^,^^24^^,^^25^ Masthoff et al^24^ found that in a study of Dutch children and adults, IgE to Cor a 9 ≥1 kU/L or Cor a 14 ≥5 kU/L in children and ≥1 kU/L in adults had a specificity of >90%.

Storage proteins have also been identified as allergens for walnut, cashew, pistachio, pecan, almond, and Brazil nut.^27^^,^^32^^,^^33^ The commercial availability of tree nut component testing varies by country. In the United States, commercially available testing toward tree nut storage proteins includes sIgE toward Ber e 1 (Brazil nut), Ana o 3 (cashew), and Jug r 1 (walnut). Testing is also available to the walnut LTP component, Jug r 3. In addition to these components, in Europe, testing is also available to additional storage proteins Ana o 2 (cashew), Jug r 2, Jug r 4, Jug r 6 (walnut), and Pis v 1, Pis v 2, Pis v 3 (pistachio). Limited studies show that sensitization to these proteins might improve diagnostic accuracy over whole extract, but additional studies are needed.^27^^,^^32^, ^33^, ^34^, ^35^

The major allergens in cow's milk are casein (Bos d 8) and beta-lactoglobulin (Bos d 5). Some studies have shown that Bos d 8 is a marker of persistent cow's milk allergy (CMA) and the best predictor of CMA.^36^ Higher levels to Bos d 8 have been suggested as a predictor for baked milk reactivity as casein is more resistant to extensive heating.^19^^,^^37^^,^^38^ A recent report of baked milk OFCs for patients with a history of CMA found no association of sIgE or casein IgE and OFC outcome.^39^ Recommendations for the optimal cutoff for Bos d 8 and unheated or baked OFCs have varied.

Ovomucoid (Gal d 1) and ovalbumin (Gal d 2) are the major allergens in hen's egg.^40^ Some evidence suggests that higher Gal d 1 IgE is associated with persistence of egg allergy.^41^ Ovomucoid IgE may be useful in predicting baked egg OFC outcomes, but there is no clear greater clinical utility compared with egg white SPT or sIgE.^42^, ^43^, ^44^, ^45^

The major wheat allergens for food allergy are gliadins and glutenins. Several studies have shown an association between omega-5-gliadin (Tri a 19) and wheat-dependent exercise-induced anaphylaxis.^46^^,^^47^ In a Japanese and Swedish population, Tri a 19 has been correlated with OFC-proven IgE-mediated wheat allergy, but the usefulness in other populations and the benefit compared with other diagnostic modalities remain unclear.^30^^,^^31^^,^^47^^,^^48^ Currently, Tri a 19 is not commercially available for IgE testing in the United States but is available in Europe.

There are very few studies on CRD and sesame allergy. Ses i 1-sIgE has been associated with clinical sesame allergy with improved specificity over sesame sIgE.^14^^,^^49^

The use of CRD in clinical practice can be determined based on patient history. A top-down, clinically driven approach to the use of CRD in clinical practice has been described by the European Academy of Allergy and Clinical Immunology.^50^ This approach starts with a thorough history and clinical examination and then SPT and/or sIgE testing. If positive, then CRD is performed. This approach could be modified based on the allergen and patient's history. The ability to distinguish clinically significant sensitization can be particularly informative for peanut or hazelnut in a birch-sensitized individual. For example, in a patient with a prior diagnosis of peanut allergy without a reaction history and known birch pollen allergy, obtaining total and component sIgE at the same time could be useful.

CRD can improve diagnostic accuracy and help stratify clinical risk, but results must be interpreted within the context of the patient's clinical history. Many components are now commercially available, but many important knowledge gaps remain. Specifically, milk or egg component testing has not consistently been shown to predict baked milk or baked egg tolerance and therefore should not be used to identify candidates for baked OFCs. CRD is relatively expensive but frequently performed as a reflex despite the fact that for most allergens, there are no well-defined cutoffs or clear utility over whole extract or SPT. The use of CRD should be individualized and universal reflex testing should be actively discouraged, because, for most patients, CRD adds little diagnostic value aside from peanut and hazelnut.^39^^,^^42^

## Specific IgE to Allergen Epitopes

Identifying the specific epitopes that IgE binds to within individual allergens can be very informative to confirm the clinical relevance of IgE sensitization and to define prognosis of specific food allergies. Different methods to detect peptide-specific IgE have been developed over time, namely using Spot membranes, microarrays, Luminex-based assays (LBA), and assays combining peptide microarray with basophil activation assays.^51^^,^^52^ The evidence of clinical utility of such tests has been recently systematically reviewed.^53^^,^^54^ The most recent studies have focused on peanut^55^, ^56^, ^57^, ^58^ and cow's milk^59^^,^^60^ allergies as well as allergies to shellfish^61^, ^62^, ^63^ and legumes.^64^, ^65^, ^66^

Using a peptide microarray, Caubet et al^60^ identified a greater intensity and broader diversity of IgE binding in children with persistent CMA, which decreased as children became tolerant. Tolerance was also associated with matched peptides recognized by IgE and IgG4. Using an LBA, a sequentially lower degree of IgE binding was found as the cow's milk–allergic patients acquired tolerance to less processed forms of cow's milk in another study.^60^ Milk-oral immunotherapy (OIT) reduced IgE but not IgG4 diversity and decreased IgE and IgG4 binding to milk peptides, with omalizumab attenuating the reduction in IgE and increasing IgG4 binding to milk peptides; however, such effect could have been due to an overall decrease in IgE levels with omalizumab. Lower IgE levels and lower diversity to milk peptides at baseline were associated with sustained unresponsiveness after OIT to cow's milk. Models based on sIgE to key epitopes combined allowed us to predict the response to treatment with 86% accuracy.

Important aspects that need to be considered when testing for IgE binding to short peptides are that it may miss some epitopes, namely discontinuous conformational epitopes, and that by narrowing from proteins to peptides one may lose the big picture. Testing for peptide-specific IgE may need to be complemented with sIgE to whole allergen components. In a recent peanut study,^55^ 7 key IgE epitope-containing peptides from Ara h 1, Ara h 2, and Ara h 3 were identified as discriminating peanut allergic from peanut-sensitized but tolerant children among peptides from all peanut allergens Ara h 1 to Ara h 11. One peptide from the peanut profilin Ara h 5 was bound more by IgE of tolerant children. The results with the microarray were validated with the results from IgE binding to the peptides when coupled to the ImmunoCAP platform. The 4 peptides from Ara h 2 proved to be diagnostically useful when combined with Ara h 2-sIgE and enhanced the diagnostic accuracy of Ara h 2-sIgE, which was already very high.

## Basophil Activation Test

The basophil activation test (BAT) is a functional assay that takes all characteristics of IgE, and possibly interfering antibodies, into account and thus has the potential to provide a result that is closer to the patients' phenotype compared with tests that quantify the levels of IgE. The diagnostic utility of the BAT and a roadmap to bring it into clinical practice have been reviewed elsewhere.^3^^,^^67^^,^^68^ The main advantage of BAT over SPT and sIgE is its high specificity, which supports the diagnosis of food allergy when BAT is positive. For instance, BAT to peanut showed a 96% specificity in a discovery cohort and a 100% specificity in a validation cohort.^10^ Applying the diagnostic cutoffs defined in this study to a large number (n = 981) of BATs of the participants in the Learning Early About Peanut Allergy (LEAP), Persistence of Oral Tolerance to Peanut (LEAP-On), and Peanut Allergy and Sensitization (PAS) studies, using the exact same methodology, confirmed the high specificity (98.5%) of BAT to diagnose peanut allergy.^69^

The best diagnostic performance for BAT was published in a recent study of CMA, where BAT to cow's milk had a 100% sensitivity and 100% specificity; however, only 41% of the children studied had IgE to cow's milk, 5% had non–IgE-mediated CMA, and 54% were neither sensitized nor allergic, which may have contributed to the excellent discriminatory ability of BAT. Similar to what was previously shown for baked milk allergy and tolerance, BAT to egg was able to distinguish different phenotypes of egg allergy in another study, in which patients reactive to baked egg showed a higher proportion of activated basophils after stimulation with egg (at the 10 and 100 ng/mL concentrations) compared with baked egg tolerant. New studies on BAT to tree nuts and sesame in children who are multisensitized to this food group are emerging. In the “Nut Cracker” study, the area under the ROC curve for BAT to tree nuts ranged between 0.78 for pecan and 0.97 for cashew.^70^ The same group reported a study of BAT to sesame that showed an area under the ROC curve of 0.86.^71^

The BATs to peanut and hazelnut have showed higher diagnostic accuracy when compared with sIgE to the respective components, namely Ara h 2 and Cor a 9/Cor a 14.^10^^,^^72^ In the Pronuts study, BAT to Ara h 2 had higher diagnostic accuracy than BAT to peanut, and in a Dutch study, stimulating the basophils with both Ara h 2 and Ara h 6 increased the sensitivity of the BAT to 79% from 72% for Ara h 2 alone and from 74% for Ara h 6 alone,^73^ suggesting that the combined use of individual allergens and the BAT could lead to a better diagnostic tool for peanut allergy. In peach allergy, BAT to Pru p 3 has also shown to be superior to BAT to peach extract, possibly due to the presence of cross-reactive allergens in the extract and the fact that Pru p 3 seems to be the primary sensitizer and the main inducer of effector cell activation in allergy to peach, at least in Southern Europe.^74^^,^^75^ After initial studies by Commins et al^76^ showing basophil activation coinciding with allergic reactions to alpha-gal during OFC to red meat, Mehlich et al^77^ studied the diagnostic utility of BAT to alpha-gal and pork kidney in identifying clinically relevant sensitization to this carbohydrate. BAT can also be used *ad hoc* to diagnose allergy to unusual allergens, such as beer or cannabis, as recently reported.^78^^,^^79^

Table III summarizes some of the cutoffs published for BAT to different foods. Given that BAT requires fresh blood and approximately 10% to 15% of individuals have nonresponder basophils (ie, basophils that do not respond to IgE-mediated stimulants such as allergen and anti-IgE but only to non–IgE-mediated stimulants), mast cell activation tests (MATs) are being researched and can overcome these limitations.^84^ The practical aspects of the BAT, for instance requiring flow cytometry and fresh blood, have led to the proposed use of BAT in clinical practice as a second- or even third-line test in patients with equivocal SPT and sIgE who would otherwise have been referred for OFC.^10^^,^^67^ This approach had 97% accuracy in a peanut study^10^ and 91% accuracy in a sesame study.^71^ This 2-step approach can reduce the number of patients tested on BAT and the number of patients requiring OFC (eg, approximately 67% reduction in the peanut study^10^), who are mainly patients with a positive BAT, who would react if they effectively underwent the OFC.

**Table III:** Examples of diagnostic cutoffs for the basophil activation test (BAT) to extracts and component allergens and their positive predictive value (PPV)

BAT	Cutoff	PPV (%)
Cow's milk^80^	>6% %CD63+ basophils	81
Ovalbumin to diagnose egg allergy^81^	≥5% %CD63+ basophils	100
Peanut^10^	≥4.78% %CD63+ basophils	100
Ara h 2 to diagnose peanut allergy^73^	≥65.19% maximal %CD63 to Ara h 2/anti-IgE	100
Sesame^71^	≥10.9% %CD63+ basophils	94
Cashew nut^70^	≥5.5% %CD63+ basophils	91
Pistachio nut^70^	≥5.8% %CD63+ basophils	74
Walnut^70^	≥6.4% %CD63+ basophils	92
Pecan^70^	≥2.7% %CD63+ basophils	75
Hazelnut^72^	CD-sens>1.7	89
Pru p 3 to diagnose peach allergy^75^	>20% CD63+ basophils	96
rTri a 19 to diagnose IgE-mediated wheat allergy^82^	>7.9% CD203c+ basophils	81
Salmon^83^	0.54 ratio of %CD203c+ basophils to allergen and anti-IgE	85

PPV was estimated from prevalence and sensitivity when not reported in the respective study.

## Resolution, Severity, and Threshold

The exact immune mechanisms as to how food allergies resolve are still not fully understood. Studies have demonstrated that some foods such as milk and egg are more commonly outgrown during childhood compared with others. The sequential use of tests over time to predict allergic resolution has been shown to be a helpful prognostic factor for future oral tolerance. Kim et al^85^ found that sIgE levels to egg and milk at the time of first reaction were a significant prognostic factor in determining future oral tolerance to these allergens. Another study found that a decrease in sIgE levels to egg and milk was significantly associated with the probability of developing clinical tolerance.^86^

Of the children who have a confirmed IgE-mediated allergy, the decision to help guide assessment of tolerance often involves confirmation via an OFC, which remains the gold standard for food allergy diagnosis. The timing of when is best to rechallenge a patient is important yet difficult to standardize in terms of management. In a multicenter study looking at the severity and threshold of peanut reactivity during OFCs, teenage children were 3 times more likely to develop anaphylaxis than younger children and there was a small but significant association between the size of peanut SPT and anaphylaxis.^87^ Santos et al^88^ reported that in a study looking at OFC to peanut, the children who had severe reactions had higher levels of peanut sIgE including the peanut components Ara h 1 and Ara h 2 compared with those with mild-to-moderate reactions. Identifying patients at higher risk of severe reactions can help avoid high-risk OFC, and the BAT has been shown to identify severe reactions of peanut-allergic children with a 97% specificity and 100% sensitivity. Patients with lower threshold of reactivity during OFC had higher basophil activation to peanut *in vitro*, which could prove to be a useful tool in identifying those patients where conducting an OFC would put them at greater risk of reacting^69^ and therefore avoidance would be appropriate. The association between BAT and threshold dose or severity of allergic reactions to peanut has been demonstrated in other studies.^88^, ^89^, ^90^

## Implications for Therapy

The importance of diagnostic testing becomes even more clear as we move into an era of food allergy treatments. There are at least 4 areas in which these implications are most obvious, certainly with more to come.

First and most obvious, it would be completely wrong to initiate treatment in a patient who is not allergic. Therefore, it is essential that the clinician be certain of the diagnosis before offering treatment. Although this may be justified even in the absence of a clinical history or OFC, for example in a patient with a peanut SPT wheal of 15 mm and an Ara h 2 level >100 kU_A_/L, for the vast majority of patients, treatment should not be considered without evidence of clinical reactivity.

Second, given that the goals of treatment at this stage for peanut OIT and epicutaneous immunotherapy (EPIT) are to prevent reactions to small, accidental exposures, it is important that treatment only be offered to those patients at risk of reacting to these minute amounts of peanut. That is, if a patient's peanut threshold without treatment is greater than 500 mg, or even the 300 mg provided in AR101, there is no reason to expect that they would benefit from treatment.^91^ So although their risk with treatment may be small, there is really no reason to undertake the expense or inconvenience of a long-term treatment.

Third, diagnostic testing without the need for repeated OFCs would be ideal as a means of measuring treatment response. Unlike OIT, where you at least know that the patient is tolerating a daily dose of peanut, this is especially important for EPIT and other treatments in development where you cannot know without OFCs if the patient is responding to treatment. This is also essential information for the patients who wish to transition from treatment to a dietary form of the food to maintain their desensitization, or for the patients who would like to know if they need to maintain treatment. Any surrogate for OFCs in this context would be of tremendous value.

Last, as new treatments are being developed, it would be ideal to be able to conduct at least phase 1 and phase 2 studies with less need, or even no need, for OFCs to both select patients for inclusion and assess efficacy.

## Recommended Clinical Approach

**Figure 1:**
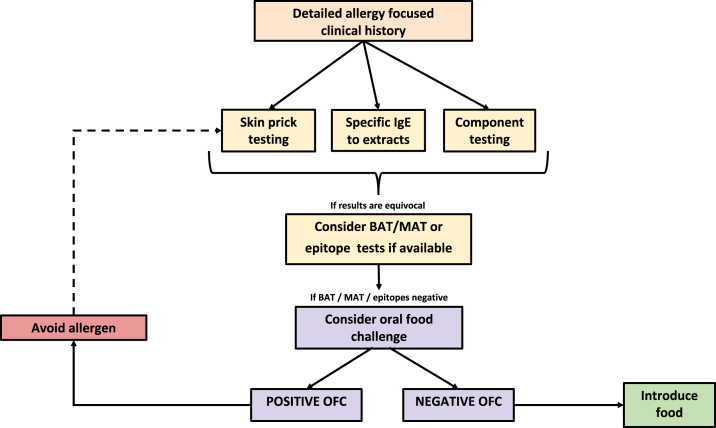
Proposed clinical approach to the diagnosis of IgE-mediated food allergy. *BAT*, Basophil activation test; *MAT*, mast cell activation test; *OFC*, oral food challenge.

Table IV summarizes the advantages and limitations of various tests that can be used to support the diagnosis of food allergy. The management of food-allergic patients can vary according to many factors—age of the child, the type of food allergen involved, and other risk factors including other atopic diseases. However, a general recommended clinical approach can be very valuable in guiding management (Figure 1).

**Table IV:** Advantages and limitations of different tests used to support the diagnosis of food allergy

Tests	Advantages	Limitations
Skin prick test	• Quick results • Results evident to patients, which can be used as an educational resource • High sensitivity, high NPV • Allows testing with fresh food	• Low specificity, low PPV (95% PPV cutoffs can increase specificity) • Risk of very rare systemic allergic reactions • Requires stopping antihistamines • Requires clear skin, free from eczema • Requires a trained professional to perform the test
Specific IgE to extracts	• Quantitative, allows us to measure exact quantity of allergen-specific IgE • Standardized methodology • High sensitivity, high NPV • *In vitro* test not affected by antihistamines • Uses serum and thus can be performed in stored samples	• Low specificity, low PPV • More expensive than SPT • Limited range of allergens available
Specific IgE to components	• High specificity (for Ara h 2 from peanut and Cor a 9/14 from hazelnut) • Differentiates between primary food allergy and clinically irrelevant IgE cross-reactivity	• Not more informative for allergenic foods other than peanut and hazelnut • Limited range of allergen components available • Sensitization profiles are patient specific and testing to single components in isolation may lead to false-negative results
Specific IgE to allergen peptides	• Certain peptides have been identified as informative to identify allergic patients (eg, peanut) • IgE to certain peptides can increase diagnostic discrimination to IgE to whole allergen (eg, Ara h 2)	• Only detects sequential epitopes • Tested in isolation (without testing to whole allergen) can lead to false-negative results
Basophil activation test	• High specificity and sensitivity • High PPV and NPV • *In vitro* test and thus no risk of allergic reactions • Does not require stopping antihistamines or clear skin	• Performed within 24 h of blood collection • Requires flow cytometry • 10%-15% nonresponders • More expensive than specific IgE or SPT
Mast cell activation test	• High specificity and PPV • Uses plasma and thus can be performed using stored samples • Provides results for individuals with nonresponder basophils • *In vitro* test and thus no risk of allergic reactions • Does not require stopping antihistamines or clear skin	• Lower sensitivity and NPV than BAT • More expensive than BAT, specific IgE, or SPT

*BAT*, Basophil activation test; *NPV*, negative predictive value; *PPV*, positive predictive value; *SPT*, skin prick test.

It is vital that a clear, careful, and detailed history is recorded at the time of presentation—be that the first index reaction or a history of multiple reactions in the past. The clarification of whether the reaction is IgE mediated is crucial in directing the investigations and management of these patients. IgE-mediated reactions usually occur within an hour of ingestion of the suspected allergen although can occur up to 4 hours later by definition. The clinical manifestations can affect various organ systems including cutaneous (ie, erythema, rash, pruritus, urticaria/hives, angioedema), respiratory (sneezing, rhinitis, coughing, wheeze, difficulty in breathing), cardiovascular (hypotension, dizziness), abdominal (pain, diarrhea, vomiting, nausea), and neurological (confusion, change in behavior, loss of consciousness), or result in life-threatening anaphylaxis.

In some cases, clinical history alone may suffice in diagnosis of IgE-mediated food allergy; however, targeted SPT and sIgE based on the clinical history to identify the pretest probability of a patient having a reaction can be used to help confirm the causative allergen(s) in more ambiguous cases. To improve this, applying 95% positive predictive cutoffs to sIgE and SPT for specific allergens can be useful. Further sIgE component testing may also be of diagnostic benefit for specific allergens, especially peanut and hazelnut (Table II). If the combination of clinical history, SPT, and sIgE and/or IgE component testing including PPV cutoffs is suggestive of a high posttest probability of a reaction occurring, an OFC is unlikely to be recommended to confirm the diagnosis as the risk of reacting would be high and unlikely to change the management of the patient. The patient would be advised to avoid the allergen and should be prescribed emergency medications.

However, if the outcome from the above tests is equivocal, BAT could be a useful tool. If BAT is highly positive, it is likely to confirm food allergy and providers could then potentially avoid an unnecessary OFC resulting in a severe or life-threatening reaction. If BAT shows nonresponsive basophils or obtaining a suitable fresh sample within the diagnostic testing time frame is not possible, the MAT could be an alternative test option. Given that both BAT and MAT are not available in most clinical settings, then proceeding to an OFC for confirmation of allergic status would be an appropriate step in confirming a food allergy diagnosis. It is also important to recognize that there will be children where their test probability is low. This may be seen in their initial test results or as a change over time but it is likely that they would benefit from having an OFC to see if they have developed tolerance. In this circumstance, the use of diagnostic cutoffs may help guide when the timing of conducting one is most appropriate and safe.

## Conclusion

In summary, the clinical history is key for an accurate diagnosis of food allergy. Allergy tests can support the diagnosis of food allergy and reduce the number of OFCs. SPT and sIgE have high sensitivity and CRD and BAT have high specificity. Combining tests can improve the accuracy of food allergy diagnosis, namely by using them in a sequential way, with CRD and BAT after detection of allergen-specific IgE with SPT or sIgE. In the cases of equivocal outcomes after combining tests or when not all of the tests are available, one needs to be prepared to conduct OFCs to confirm or exclude the diagnosis of food allergy.
